# Overexpression of RPN2 suppresses radiosensitivity of glioma cells by activating STAT3 signal transduction

**DOI:** 10.1186/s10020-020-00171-5

**Published:** 2020-05-13

**Authors:** Changyu Li, Haonan Ran, Shaojun Song, Weisong Liu, Wenhui Zou, Bei Jiang, Hongmei Zhao, Bin Shao

**Affiliations:** 1 Neurosurgery, Hainan Cancer Hospital, Haikou, China; 2 Radiotherapy Department, Hainan Cancer Hospital, Haikou, China; 3 Head and Neck Surgery, Hainan Cancer Hospital, Haikou, China; 4 Hematology Department, Hainan Cancer Hospital, Haikou, China; 5 Clinical Pharmacy Department, Hainan Cancer Hospital, Haikou, China; 6 Neurosurgery, The First Affiliated Hospital of Harbin Medical University, No.199 Dazhi Street, Nangang District, Harbin, 150001 Heilongjiang China

**Keywords:** GBM, RPN2, STAT3, Signal transduction

## Abstract

**Background:**

Radiation therapy is the primary method of treatment for glioblastoma (GBM). Therefore, the suppression of radioresistance in GBM cells is of enormous significance. Ribophorin II (RPN2), a protein component of an N-oligosaccharyl transferase complex, has been associated with chemotherapy drug resistance in multiple cancers, including GBM. However, it remains unclear whether this also plays a role in radiation therapy resistance in GBM.

**Methods:**

We conducted a bioinformatic analysis of *RPN2* expression using the UCSC Cancer Genomics Browser and GEPIA database and performed an immunohistochemical assessment of RPN2 expression in biopsy specimens from 34 GBM patients who had received radiation-based therapy. We also studied the expression and function of RPN2 in radiation-resistant GBM cells.

**Results:**

We found that *RPN2* expression was upregulated in GBM tumors and correlated with poor survival. The expression of RPN2 was also higher in GBM patients with tumor recurrence, who were classified to be resistant to radiation therapy. In the radiation-resistant GBM cells, the expression of RPN2 was also higher than in the parental cells. Depletion of RPN2 in resistant cells can sensitize these cells to radiation-induced apoptosis, and overexpression of RPN2 had the reverse effect. Myeloid cell leukemia 1 (MCL1) was found to be the downstream target of RPN2, and contributed to radiation resistance in GBM cells. Furthermore, STAT3 was found to be the regulator of MCL1, which can be activated by RPN2 dysregulation.

**Conclusion:**

Our study has revealed a novel function of RPN2 in radiation-resistant GBM, and has shown that MCL1 depletion or suppression could be a promising method of therapy to overcome the resistance promoted by RPN2 dysregulation.

## Background

Glioblastomas (GBM) are tumors of the central nervous system and result in 1.2 ~ 1.4 × 10^4^ deaths in the U.S. alone every year (Hess et al., 2004). The existing treatment methods are usually limited to surgical excision, radiation, and temozolomide chemotherapy (Stupp et al., 2005). Despite these aggressive treatments, survival rates after treatment remain low, at an average of around 12–15 months (Preusser et al., 2011). It is thought that GBM tumors can obtain chemo and radiation therapy resistance by stimulating DNA repair systems or by initiating changes to the cell cycle and apoptosis (Mirimanoff et al., 2006; Mrugala & Chamberlain, 2008). Resistance to chemotherapy has been widely investigated and has mostly focused on the expression of *MGMT* (0–6 methylguanine-DNA Methyltransferase) (Perazzoli et al., 2015). However, the fundamental mechanisms underlying radiotherapy resistance and its generation are still unclear. Radiation therapy remains a primary method of treatment for GBM (Ghotme et al., 2017), and therefore the reduction of radioresistance in GBM cells and therapeutic targets is of huge significance.

Ribophorin II (RPN2) is a protein component of an N-oligosaccharyl transferase complex, the downregulation of which can trigger apoptosis in human breast cancer cells resistant to docetaxel., and its silencing confers sensitivity of the tumor to cisplatin treatment (Honma et al., 2008). In addition, gastric cancers with high RPN2 expression have exhibited dramatically higher recurrence rates and lower 5-year survival rates relative to those with low expression (Fujimoto et al., 2017). These observations suggest that RPN2 expression could serve as a predictive biomarker for chemotherapy resistance. In a recent study, RPN2 was reported to be modulated by circNFIX, and promoted GBM tumor growth in vivo and in vitro (Ding et al., 2019). However, the correlation of RPN2 expression and radiotherapy resistance in GBM remains unknown.

This study explored the function of RPN2 in radioresistant GBM, and found that its high expression contributes to the tolerance of GBM to radiotherapy. The dysregulation of RPN2 led to abnormal myeloid cell leukemia 1 (MCL1) expression through the promotion of STAT3 transcription activity. Our study, therefore, provides a new target to overcome radioresistance in GBM therapy.

## Methods

### Bioinformatics analysis

The abnormal expression of *RPN2* and *MCL1* was investigated through the UCSC Cancer Genomics Browser (https://xena.ucsc.edu/welcome-to-ucsc-xena/) and GEPIA online database (http://gepia.cancer-pku.cn/).

### Patient samples and cell culture

GBM samples were taken from 34 patients admitted to the First Affiliated Hospital of Harbin Medical University. These GBM patients had all received radiation therapy, with 12 patients experiencing GBM recurrence. The corresponding brain samples were harvested and preserved at − 80 °C. Informed consent was obtained from all participants, and the study was approved by the Ethics Committee of the First Affiliated Hospital of Harbin Medical University.

The normal glioma cell lines (U87, T98, U251, U-118MG and A172) and astrocyte cell line (HA) were provided by BeNa Culture Collection (Beijing, China). These cells were cultivated in DMEM (Sigma, St. Louis, MO, USA) with 10% FBS at 37 °C under 5% CO_2_.

### Radiotherapy

Radiotherapy was conducted in the Radiotherapy Oncology department of the First Affiliated Hospital of Harbin Medical University, using a Varian 2100C linear accelerator (dose, 5 Gy; dose rate, 5 Gy/min). The cells were seeded in a 12-well plate and preserved under adjustable conditions for 1 day, and subsequently treated with radiation, and cultivated again under identical conditions for 1 day more.

### Clonal radioresistant cell generation

A172 and U87 cells were seeded in culture plates (100 mm) and treated with a 1 Gy radiation dose until an accumulated dose of 5 Gy was reached. All dissociated cells were recovered using cloning cylinders (Sigma Aldrich) and seeded in a 96-well plate. Once proliferating, these cells were transferred to a 12-well plate, and subsequently a 6-well plate. Colonel radioresistant cells were then obtained.

### Cell transfection

U87 cells were inoculated in 12-well plates (1 × 10^5^ cells/well). *RPN2*, *MCL1*, and *STAT3* siRNA or control siRNA (GenePharma, Co., Ltd., Shanghai, China) were transfected into cells through Lipofectamine™ 2000 (Invitrogen, Shanghai, China) at 50–60% confluency. Two days later, the resulting cells were harvested and preserved for future use. The siRNA sequences involved were: *RPN2*, 5′-GGCCACUGUUAAACUAGAACA-3′; *MCL1*, 5′-CGCCGAAUUCAUUAAUUUAUU-3′; *STAT3*, 5′-CUUCAGACCCGUCAACAAA-3′. The negative (mock) siRNA sequence involved was 5′-ACGCCUCCCGAACGUTTUUCUUGUCGUC-3′. The pcDNA-V5-*RPN2* was constructed by inserting the *RPN2* cDNA into pcDNA™3.1/V5-His TOPO vector (Thermofisher, Waltham, MA. USA) according to the manufacturer instructions.

### qPCR

RNA was isolated using TRIzol (Thermo Fisher Scientific) and quantified using a NanoDrop 2000 (Thermo Fisher). RNA was treated with RNase R (Geneseed, Guangzhou, China) to achieve circRNA purification. The cDNA was prepared from 0.5 μg RNA using a Prime-Script RT reagent kit (TaKaRa, Dalian, China) or TaqMan miRNA Reverse Transcription Kit (Applied Biosystems, FosterCity, CA, USA). qPCR was conducted using a PCR System (Bio-Rad). The following were primer sequences involved: *RPN2* (Forward, 5′- AGGAAGTGGTGTTTGTTGCC-3′; Reverse, 5′-CAGTCGAGGGAGCTTCTTC-3′); *MCL1* (5′-AAAGCCTGTCTGCCAAAT-3′ and reverse primer 5′-CCTATAAACCCACCACTC-3′); and *GAPDH* (Forward, 5′-GAATGGGCAGCCGTTAGGAA-3′; Reverse, 5′-AAAAGCATCACCCGGAGGAG-3′).

### Western blot (WB)

Western blot analysis was conducted to isolate the target proteins using RIPA buffer (Beyotime) containing protease inhibitors. The protein concentrations were determined using a BCA Kit. Subsequently, the same amounts (0.02 mg) of protein were treated with boiled water and isolated using the SDS-PAGE technique. The SDS-PAGE gels were subsequently transferred to 0.45 μm PVDF membranes (Millipore, Billerica, MA, USA), which were exposed to TBST containing 5% fat-free milk for 60 min and cultivated with RPN2, MCL1, BCL2, BAX, BIM (Abcam, Cambridge, MA, USA), NOXA, PUMA, cleaved caspase-3 (CASP3), STAT3, p-STAT3 and β-actin (ACTB) (Cell Signaling, Danvers, MA, USA) antibodies at 4 °C overnight. After washed with TBST for 3 times, and the membranes were cultivated with 2nd antibody (Abcam) diluted at 1:1000 for 120 min at room temperature.

### Immunohistochemistry and immunofluorescence staining

GBM tissues were fixed in 10% formalin followed by paraffin embedding. Immunohistochemistry (IHC) was performed on 5 μm tissue sections using the antibodies of RPN2, MCL1 (Abcam), p-STAT3 (Cell Signaling) according to manufacturer’s instructions. The results of the IHC analysis were taken with a digital slide scanning system (Pannoramic Scan, 3DHISTECH Ltd.). The mean density (IOD/area) were quantified by Image J software (NIH, USA). The immunofluorescence staining of STAT3 and nuclear labeling with DAPI were performed as previously described (Bhattacharya et al., 2005).

### MTS and apoptosis assay

Cells (5000) were inoculated in 96-well plates and cultivated at 37 °C overnight in medium supplemented with 10% FBS. Following this, cell viability was determined using a cell proliferation kit (Promega, Madison, WI, USA). Each process was conducted in parallel at least three times, on three different occasions. The apoptosis analysis was conducted by using Hoechst33258 staining as described in the previous study (Chen et al., 2014). The results were expressed as average ± SD.

### Statistical analysis

The experiments were conducted no less than three times, and the results presented as average ± SD. Differences among groups were assessed using a Tukey test and *t*-test or with ANOVA, analyzed through Prism 7 (GraphPad Inc., La Jolla, CA, USA). Patients’ overall survival (OS) was obtained and evaluated using a K-M plot and log-rank test, respectively.

## Results

### Abnormal expression of RPN2 is correlated to poor survival of GBM patients

**Fig. 1:**
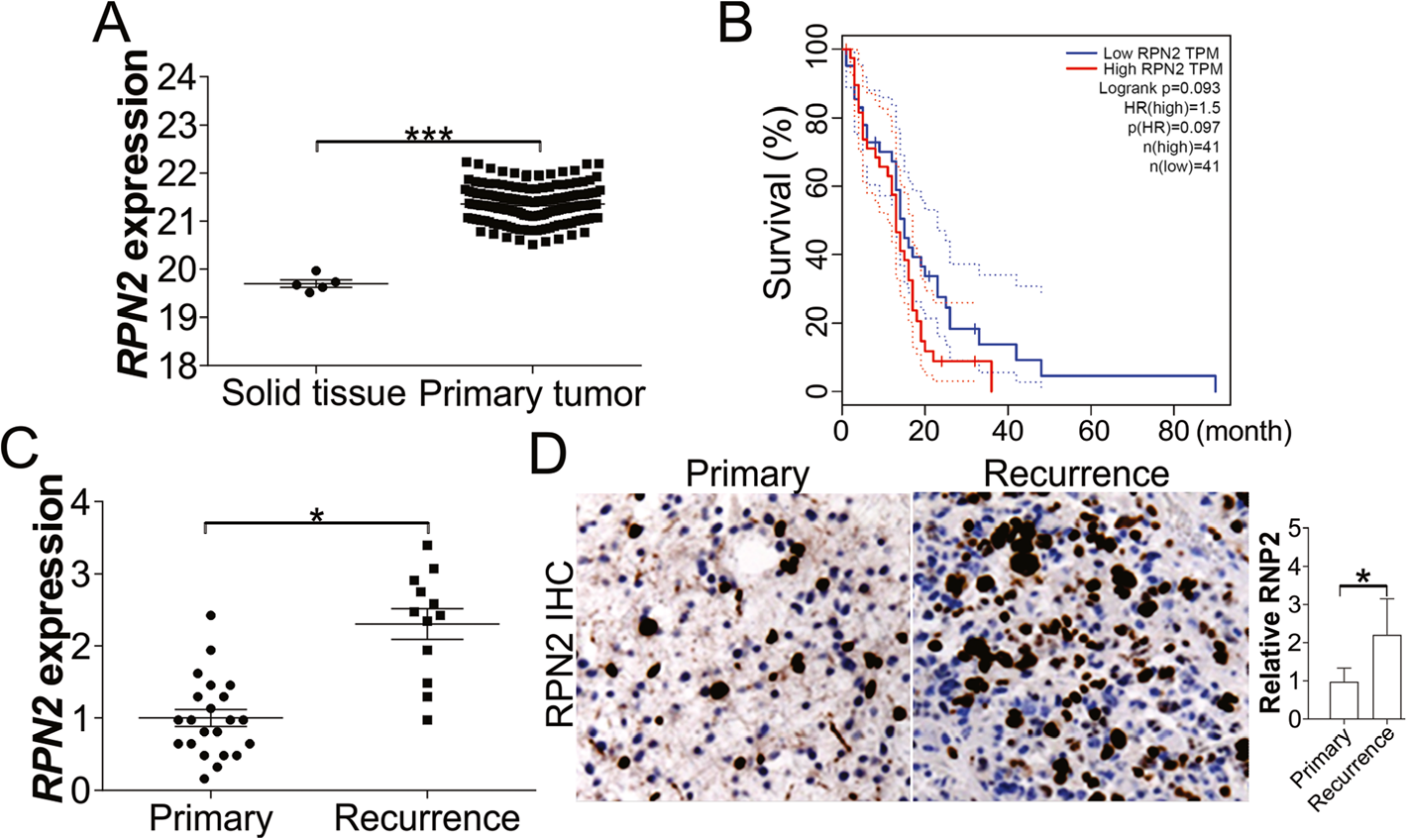
High RPN2 expression in GBM is correlated to poor survival and recurrence. **a** The mRNA expression of *RPN2* in solid tissue and primary GBM tumor based on database from cancer browser (https://xenabrowser.net/heatmap/). **b** The correlation of *RPN2* expression with GBM patient survival based on the database of GEPIA (http://gepia2.cancer-pku.cn/#index). **c** The mRNA expression of *RPN2* in 34 GBM patients with radiation therapy history. 12 of them had GBM recurrence. **d** The IHC staining of RPN2 in primary and recurrence GBM tumors. Left, the representative pictures; right, the histological analysis of RPN2 expression by Image J. *, *p* < 0.05; ***, *p* < 0.001

To study the role of RPN2 in GBM radiotherapy, we first determined the expression of *RPN2* in GBM by analyzing mRNA data in the Cancer Genome Atlas (TCGA) database. TCGA RNA-Seq data showed a marked upregulation of *RPN2* in GBM tumor tissues compared to normal control tissues (Fig. 1a, b). Our analysis of GEPIA data indicated that high expression of *RPN2* is associated with poorer survival in GBM patients, which strongly suggests that abnormal expression of *RPN2* plays an important role in GBM. Since radiotherapy is one of the primary treatment methods for GBM, we then analyzed *RPN2* expression in different groups of GBM patients. We analyzed RPN2 expression in tissue samples obtained from 34 GBM patients who had received radiation treatment. Among these 34 patients, 12 patients had experienced GBM recurrence and were grouped as radioresistant patients. We then compared RPN2 expression levels in tumor samples from these 34 patients, with or without GBM recurrence. We found that the patients with tumor recurrence had higher RPN2 expression at the mRNA and protein level (Fig. 1c, d). Our data suggests that high RPN2 expression may contribute to radioresistance in GBM.

### RPN2 expression is higher in radioresistant GBM cells

**Fig. 2:**
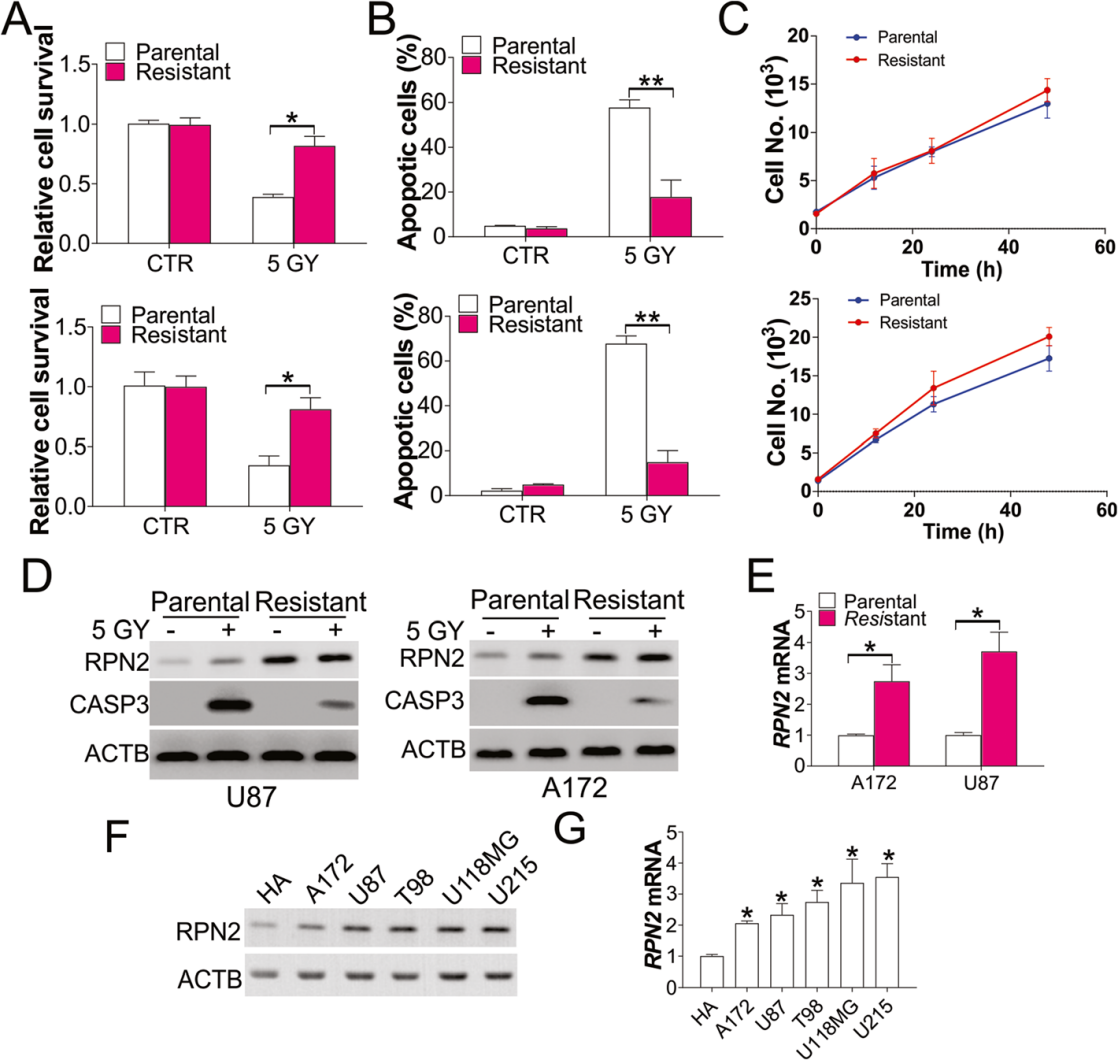
RPN2 was upregulated in radioresistant GBM cells. **a** The survival of parental and radioresistant U87 (upper) and A172 (lower) cells treated with 5 GY radiation. **b** The apoptosis of parental and radioresistant U87 (upper) and A172 (lower) cells treated with 5 GY radiation. **c** The growth of parental and radioresistant U87 (upper) and A172 (lower) cells. **d** The expression of RPN2 and CASP3 in parental and radioresistant U87 (left) and A172 (right) cells treated with 5 GY radiation. **e** The mRNA level of RPN2 in parental and radioresistant U87 and A172 cells. **f, g** The protein (**f**) and mRNA (**g**) level of RPN2 in the indicated cells. Each experiment was performed for 3 times. *, *p* < 0.05; **, *p* < 0.01

To study the role of RPN2 in radiotherapy resistant GBM, we generated radioresistant GBM cells by subjecting U87 and A172 cells lines to five successive rounds of radiation. We found that the 5 Gy radiation resulted in less cell death in resistant A172 and U87 cells, as analyzed by MTT assay (Fig. 2a). Apoptosis is the major cell death induced by radiation (Stupp et al., 2005). Therefore, we investigated the radiation triggered apoptosis in U87 and A172 cells using Hoechst 33258 staining, and observed that apoptosis was higher in parental cells than in the resistant cells (Fig. 2b). However, these cells did not display any differences in cell proliferation (Fig. 2c). We then analyzed the apoptotic signal in parental and resistant cells by analyzing cleaved CASP3 expression, and found that cleaved CASP3 was induced in A172 and U87 parental cells, while compromised in resistant cells (Fig. 2d). We then investigated the RPN2 expression in these cells and observed that resistant cells exhibited higher expression at the protein and mRNA level (Fig. 2d, e). Furthermore, the expression of RPN2 in GBM cells was also higher than that in the normal astrocyte cell line (HA) (Fig. 2f, g). Therefore, our results suggest that high expression of RPN2 could be correlated with radioresistance in GBM.

### Expression of RPN2 contributes to radioresistance in GBM

**Fig. 3:**
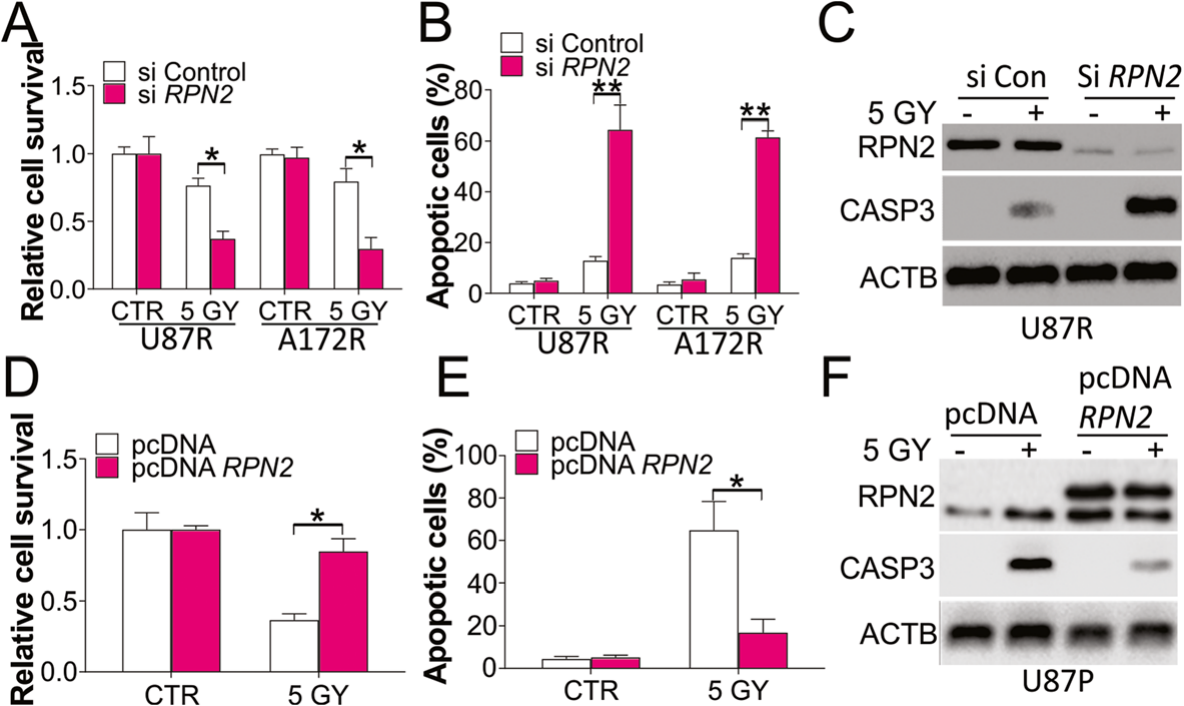
Abnormal expression of RPN2 leads to radioresistance in GBM cells. **a** U87 and A172 resistant cells transfected with control or *RPN2* siRNA were treated with 5 GY radiation. The survival was analyzed by MTS assay. **b** The apoptosis of U87 and A172 resistant cells treated as in (**a**). **c** The expression of RPN2 and CASP3 in U87 resistant cells treated as in (**a**). **d** U87 parental cells transfected with control or *RPN2* plasmids were treated with 5 GY radiation. The survival was analyzed by MTS assay. **e** The apoptosis of U87 parental cells treated as in (**d**). **f** The expression of RPN2 and CASP3 in U87 parental cells treated as in (**d**). Each experiment was performed for 3 times. *, *p* < 0.05; **, *p* < 0.01

To confirm whether high expression of RPN2 is necessary for radioresistance in GBM, siRNA-mediated knockdown of *RPN2* was carried out in U87 and A172 resistant cells. It was observed that RPN2 deprivation re-sensitized the U87 and A172 resistant cells to 5 Gy radiation induced cell death (Fig. 3a). In terms of apoptosis, absence of *RPN2* enhanced radiation induced apoptosis (Fig. 3b, c). Conversely, enhanced expression of *RPN2* in U87 parental cells had the opposite effect on radiation induced cell death, including the suppression of cell loss (Fig. 3d), prevention of apoptosis (Fig. 3e), and reduction of CASP3 cleavage (Fig. 3f). Therefore, our data indicates that RPN2 expression contributes to radioresistance in GBM cells.

### RPN2 regulated the MCL1 expression in radio-resistant GBM cells

**Fig. 4:**
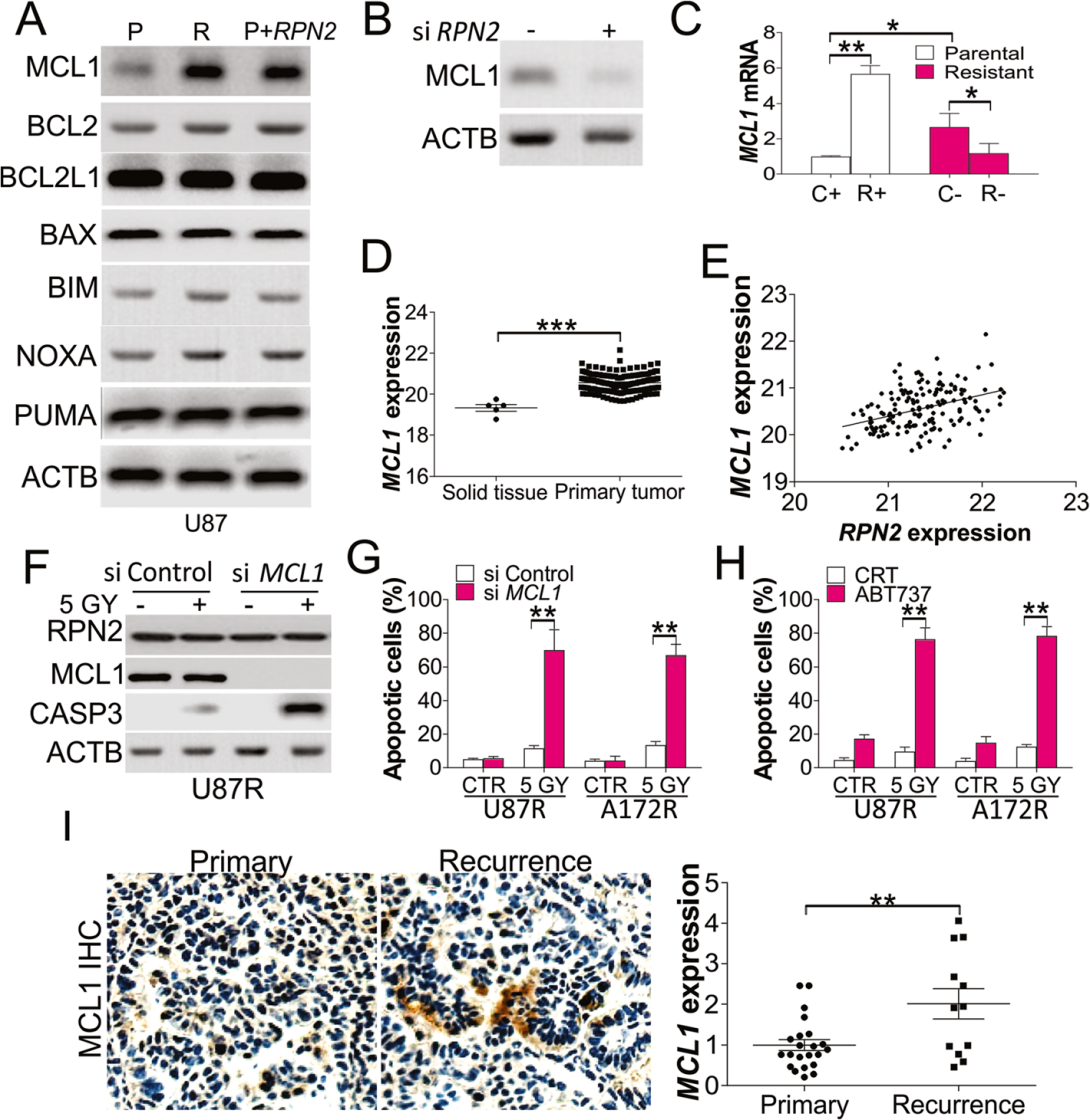
RPN2 promotes MCL1 expression to mediate the radioresistance in GBM cells. **a** The expression of Bcl-2 family proteins in U87 parental, resistant cells, or U87 parental cells with RPN2 overexpression. **b** The expression of MCL1 in U87 resistant cells transfected with *RPN2* siRNA. **c** The mRNA level of *MCL1*. C+: pcDNA transfection; R+: pcDNA *RPN2* transfection; C-: si control transfection; R-: si *RPN2* trasnfection. **d** The expression level of *MCL1* in GBM tumors based on the database of cancer browser (https://xenabrowser.net/heatmap/). **e** The pearson correlation of *RPN2* and *MCL1* from database of cancer browser. *R*^2^ = 0.1502, *p* < 0.001. **f** U87 resistant cells transfected with *MCL1* siRNA were treated with 5 GY radiation. The expression of RPN2, MCL1 and CASP3 was analyzed by western blot. **g** The apoptosis of U87 and A172 resistant cells treated as in (**f**). **h** The apoptosis of U87 and A172 resistant cells treated with ABT-737 (5 μM) in combination with 5 GY radiation. **i** The IHC staining of MCL1 in primary and recurrence GBM tumors. Left, the representative pictures; right, the histological analysis of MCL1 expression by Image J. Each experiment was performed for 3 times. *, *p* < 0.05; **, *p* < 0.01; ***, *p* < 0.001

We then investigated the mechanisms underlying the role of RPN2 in GBM radioresistance. The expression of Bcl-2 family proteins is critical for radiation induced apoptosis, and the dysregulation of these proteins usually leads to radioresistance. The expression of several Bcl-2 family proteins was assessed in U87 parental, resistant cells, and U87 parental cells with pcDNA-*RPN2* transfection. The western blot revealed that only MCL1 was upregulated in U87 resistant cells, and in U87 parental cells with *RPN2* overexpression (Fig. 4a). However, other Bcl-2 family proteins, including BCL2, BCL2L1, BAX, BIM, NOXA, and PUMA, did not show any changes (Fig. 4a). In contrast, depletion of *RPN2* by siRNA suppressed MCL1 expression in U87 resistant cells (Fig. 4b). *MCL1* mRNA levels were also consistently upregulated in U87 parental cells with RPN2 overexpression, and downregulated in RPN2 knockdown U87 resistant cells (Fig. 4c). To further confirm the relationship between RPN2 and MCL1, we analyzed *MCL1* expression in the Cancer Genome Atlas (TCGA) database, and found that *MCL1* expression was also higher in GBM primary tumors (Fig. 4d), and positively correlated with the expression of RPN2 (Fig. 4e). To further study the role of MCL1 dysregulation in radioresistant GBM, siRNA-mediated knockdown of *MCL1* was carried out by transfecting U87 and A172 resistant cells. Silencing of *MCL1* had a similar effect to that of *RPN2* knockdown, which enhanced the expression of CASP3, and nuclear fragmentation caused by radiation (Fig. 4f, g). However, absence of *MCL1* did not affect the expression of RPN2 (Fig. 4f). Similarly, pre-treatment with an MCL1 inhibitor, ABT-737, resulted in a similar effect, sensitizing the radioresistant U87 and A172 cells to radiation triggered apoptosis (Fig. 4h). The bliss synergy score of ABT737 with irradiation in these two cells were 2.09 and 1.65, respectively. The histological analysis of MCL1 expression by IHC revealed that MCL1 expression in the recurrent GBM was also higher than the primary tumors (Fig. 4i). Taken together, these findings show that RPN2 promotes radioresistance in GBM by upregulating *MCL1* at the mRNA level.

### RPN2 modulated MCL1 expression by stimulating STAT3

**Fig. 5:**
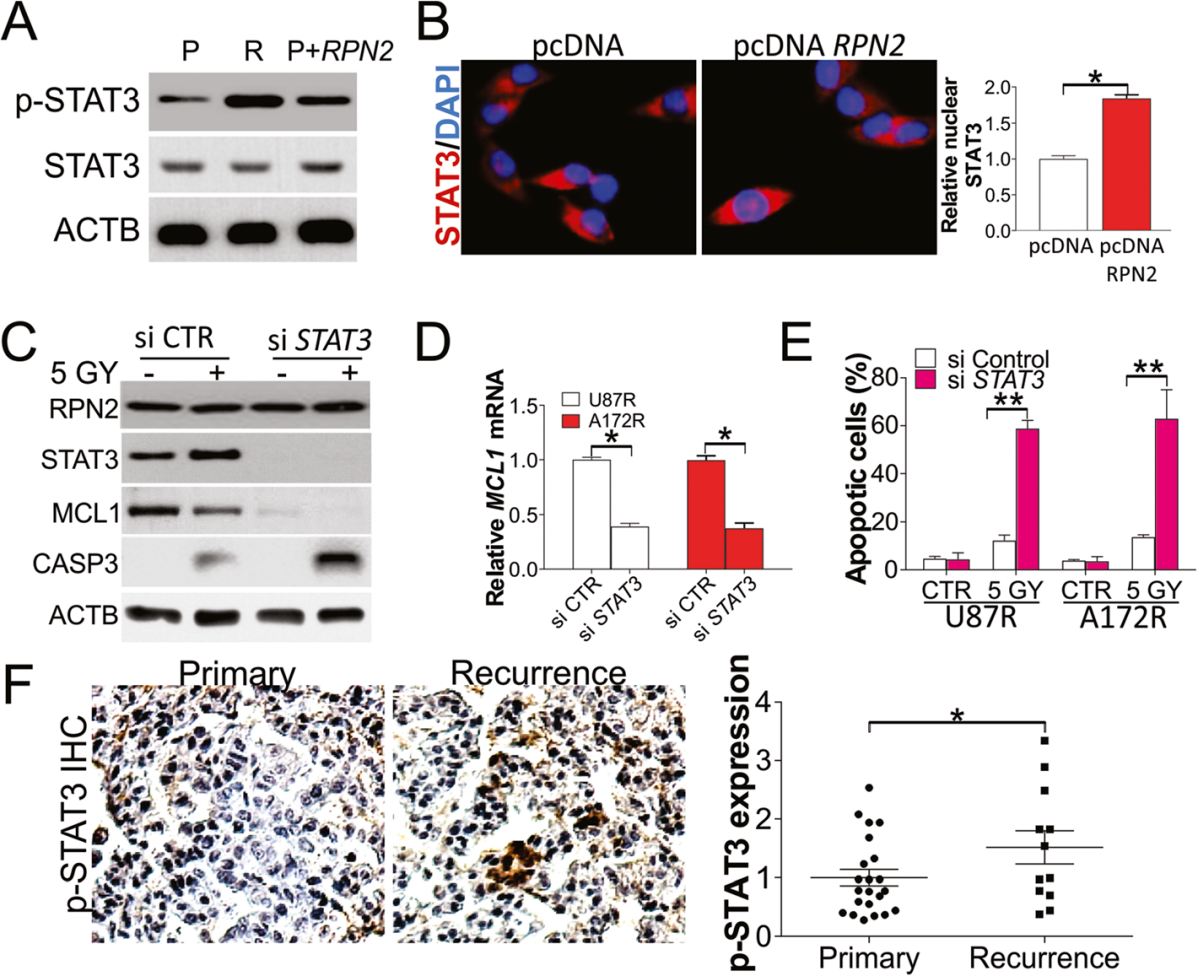
RPN2 mediates MCL1 expression by activate STAT3. **a** The expression of p-STAT3 and STAT3 in U87 parental, resistant cells, or U87 parental cells with *RPN2* overexpression. **b** The immunofluorescence staining of STAT3 in U87 cells transfected with *RPN2* plasmid. Left, the representative pictures; right, the nuclear translocation of STAT3 was analyzed by Image J. **c** U87 resistant cells transfected with *STAT3* siRNA were treated with 5 GY radiation. The expression of RNP2, MCL1, STAT3, and CASP3 was analyzed by western blot. **d** The mRNA level of *MCL1* in U87 and A172 resistant cells transfected with *STAT3* siRNA. **e** The apoptosis of U87 and A172 resistant cells treated as in (**c**). **f** The IHC staining of p-STAT3 in primary and recurrence GBM tumors. Left, the representative pictures; right, the histological analysis of p-STAT3 expression by Image J. Each experiment was performed for 3 times. *, *p* < 0.05; **, *p* < 0.01

In the next stage of this study, we assessed how RPN2 modulates the expression of MCL1 in radioresistant GBM. Previous studies have shown that MCL1 expression is regulated via STAT3 phosphorylation (Bhattacharya et al., 2005), which was recently reported to be activated by RPN2 (Ni et al., 2020). We therefore studied STAT3 activation levels upon RPN2 overexpression. As expected, RPN2 overexpression improved STAT3 phosphorylation levels without influencing its expression (Fig. 5a). And the STAT3 phosphorylation also increased the U87 resistant cells when compared with the parental cells (Fig. 5a). Furthermore, overexpression of RPN2 also enhanced the nuclear translocation of STAT3 (Fig. 5b), suggesting that RPN2 expression activates STAT3. To determine the role of STAT3 in RPN2-triggered MCL1 upregulation, we depleted *STAT3* in U87 and A172 resistant cells, and found that silencing of *STAT3* inhibited MCL1 expression at the protein and mRNA level (Fig. 5c, d). Silence of *STAT3* did not affect the expression of RPN2 (Fig. 5c). Depletion of *STAT3* sensitized U87 resistant cells to radiation induced apoptosis (Fig. 5c, e). Moreover, the phosphorylation of STAT3 was higher in the recurrent GBM tissues than the primary tumors (Fig. 5f). These findings confirm that the STAT3 pathway is associated with RPN2-modulated MCL1 expression in radioresistant GBM cells.

## Discussion

**Fig. 6:**
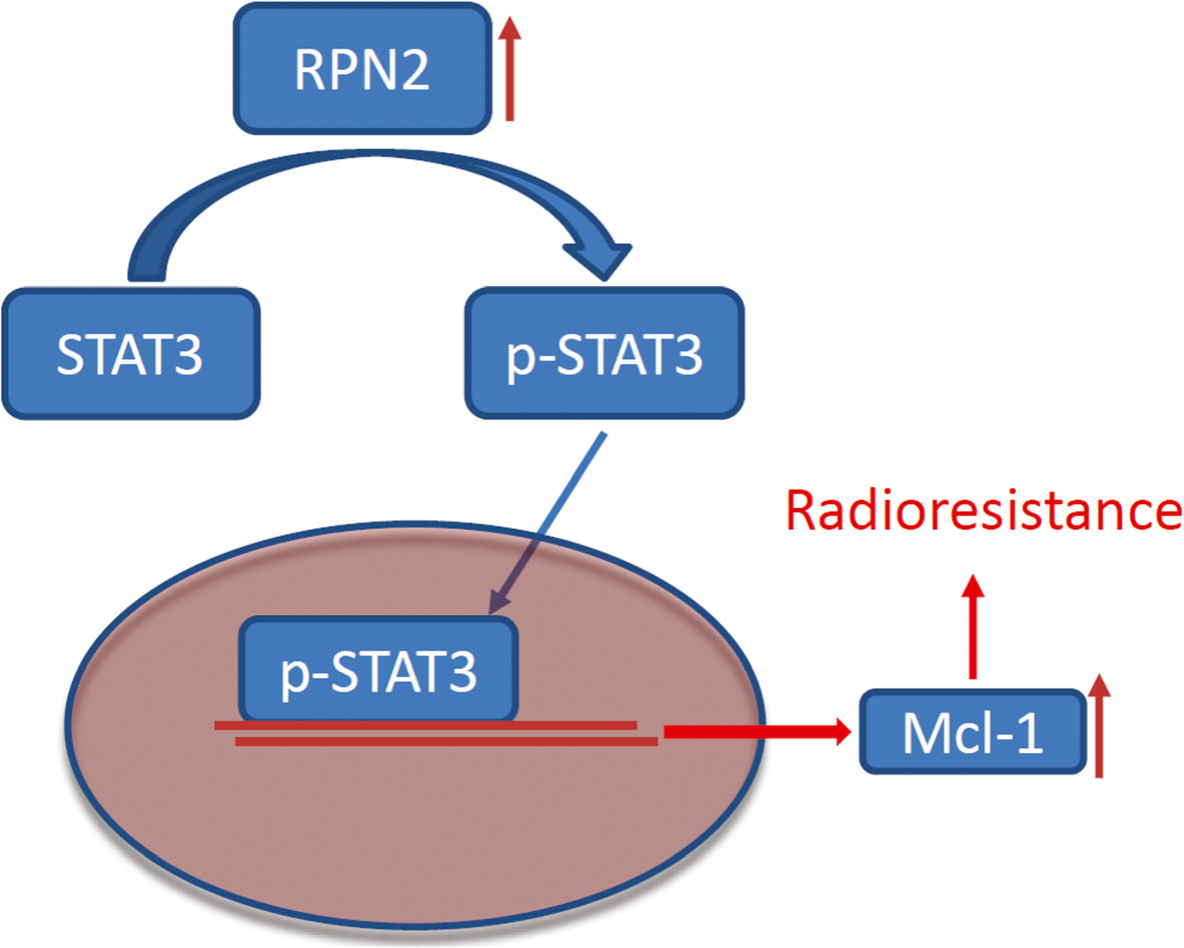
The graphic summary of RPN2/STAT3/MCL1 pathway

The *RPN2* gene has been localized to chromosome 20ql2–13.1, a region that is often compromised in patients who develop malignant myeloid tumors (Davis et al., 1984; Loffler et al., 1991). Besides its correlation with myeloid disturbance, RPN2 has also been identified as an indicator of prognosis in human pancreatic and breast cancer (Kaushal et al., 2012; Zhu et al., 2012). It has also been associated with the resistance of tumor cells to chemotherapeutic drugs in the breast (Honma et al., 2008) and ovarian (De Souza et al., 2011) cancers, and esophageal squamous cell carcinomas (Kurashige et al., 2012). Moreover, shRNAs-triggered *RPN2* silencing has been shown to suppress lung tumor formation and increase cancer cell sensitivity to cisplatin (Fujita et al., 2015). In another study, RPN2 mediated chemo-sensitivity was mediated by P-gp protein expression, which was downregulated after cisplatin treatment in gastric cancer cells without RPN2 (Yuan et al., 2015). However, the activity of RPN2 in response to different doses of ionizing radiation has not been investigated. This study has revealed a strong correlation between an increase in RPN2 expression and radioresistance in GBM in vitro and in vivo. We found that abnormal RPN2 expression promotes MCL1 expression by stimulating STAT3, thereby promoting radioresistance (Fig. 6).

The activation of STAT3 plays a vital role in mediating anti-apoptosis genes to suppress cell apoptosis, including *MCL1* (Bhattacharya et al., 2005). It was reported that activation of STAT3 suppressed cell apoptosis, and promoted cell migration and invasion, cell cycle arrest by modulating several genes’ expression in liver, ovarian and colon cancer (Bhattacharya et al., 2005; Chung et al., 1997; Huang et al., 2019). In our study, we found that RPN2 overexpression enhanced the phosphorylation levels STAT3 and promoted its nuclear translocation (Fig. 5), suggesting the activation of STAT3 by enhanced RPN2 expression. Consistent with our study, the activation of STAT3 by RPN2 was found in other types of cancers, including colon carcinoma, hepatocellular carcinoma (Huang et al., 2019; Bi & Jiang, 2018). RPN2 was reported to regulate colon cancer cell proliferation through mediating the glycosylation of EGFR, which affectes the EGFR/ERK signaling pathways (Li et al., 2017). Since EGFR/ERK can directly phosphorylate and activate STAT3 (Chung et al., 1997), we hypothesized that RPN2 might modulate the activity of STAT3 by EGFR glycosylation. However, the exact mechanism still needs further investigation.

Apoptosis triggered by DNA damage is the primary mechanism of action in radiotherapy (Norbury & Zhivotovsky, 2004). Abnormal Bcl-2 family protein expression is frequently detected in multiple cancers (Davids & Letai, 2012), and contributes to radio-resistance both in vitro and in vivo (Chen et al., 2018). Radioresistance may also result from perturbations in homologous recombination repair and DNA double-strand break repair, by error-prone and alternative end-joining. Furthermore, genetic and pharmacologic targeting of MCL1 or BCL2L1 has been shown to effectively improve the sensitivity of cancer cells to radiotherapy (Chen et al., 2018). The complicated molecular interplay of pro-survival and pro-death Bcl-2 members determinants cell survival and apoptosis rate. Alterations in this balance resulting from responses to survival stimuli is only just starting to be understood. Lopez et al displayed that apoptosis triggered by DNA damage occurs when MCL1 and BCL2L1 are suppressed (Lopez et al., 2010). Fujita et al confirmed RPN2 facilitated cell proliferation and repression of apoptosis by modulating BAX/BCL2 in non-small cell lung cancer (Fujita et al., 2015). However, we did not find that the expression of BCL2 changed with RPN2 expression in this study. Instead we found that MCL1 expression was up-regulated in radioresistant and RPN2 overexpressed GBM cells, and appears to contribute to the tolerance of radiation. Repressing MCL1 may play clinical role in treating diverse radioresistant tumors, although the exact pharmacological mechanism of MCL1 suppression has not yet been established. Some pan-Bcl-2 inhibitors were prepared, such as ABT-737, and can suppress tumor formation and survival (Davids & Letai, 2012; Thomas et al., 2013). Here we show that administration of ABT-737 can overcome the radioresistance in RPN2 overexpressed GBM cells, suggesting that inhibition of MCL1 could be a therapy strategy for GBM patients with abnormal tumor RPN2 expression.

## Conclusions

In conclusion, this study has shown an obvious distinction in overall survival between RPN2 high expression and low expression GBM patients, as well as a correlation between RPN2 expression and the effectiveness of radiation. This study is the first to indicate that RPN2 expression may serve as a predictive biomarker for radiation therapy in GBM patients.

## Data Availability

Not applicable.

## Abbreviations

GBM: Glioblastoma
RPN2: Ribophorin II
MCL1: Myeloid cell leukemia 1
WB: Western blot
OS: Overall survival
TCGA: The Cancer Genome Atlas
